# Editorial: Methods in Performance Science

**DOI:** 10.3389/fpsyg.2024.1504498

**Published:** 2024-10-23

**Authors:** Pil Hansen, Bettina E. Bläsing, Aaron Williamon

**Affiliations:** ^1^School of Creative and Performing Arts, University of Calgary, Calgary, AB, Canada; ^2^Department of Music and Movement in Rehabilitation and Pedagogy in Disability, Faculty of Rehabilitation Sciences, Technical University Dortmund, Dortmund, North Rhine-Westphalia, Germany; ^3^Royal College of Music, London, United Kingdom

**Keywords:** research methods–psychology, performing arts psychology, sports science, Performance Science, research ethics, practice-based research, transdisciplinary research

This Research Topic contributes research, methods, and perspectives to the Methods in Psychology series. The series aims to highlight developments in scientific techniques and methods used to investigate important questions. The present Research Topic focusses on the advantages and limitations of methodologies and applications in Performance Science Research, including relevant ethical, interdisciplinary and practice-based perspectives.

Performance Science is a multidisciplinary field in psychology that aims to understand, support and enhance human performance across a wide range of disciplines. This aim is pursued within sustainable development goals pertaining to education, health, economic growth and equity. The editors have all engaged extensively with the complex methodological questions that arise when working on such goals across disciplines, understanding that invitations to propose answers, like this Research Topic, are essential for the advancement of Performance Science (Williamon et al., 2021; Hansen, 2017, 2023; Bläsing et al., 2019).

Of the seven articles included here, four focus on methods for the performing arts domain. Two of these explore interdisciplinary solutions to methodological problems.

Hansen argues that the lack of diversity in psychology research samples can be harmful and is associated with norms for research methods and ethics. She suggests that performing arts psychology provides an opportunity to model solutions through transdisciplinary study designs, combining empirical methods from psychology with practice-based methods from the arts, while situating research in diverse communities with awareness of both relational and procedural ethics. Approaching this challenge from a reversed perspective, Shaughnessy et al. address the specific problem of measuring empirically the engagement of youth on the autism spectrum in practice-based, participatory art interventions. As a solution, their team developed and applied a Participatory arts Play Framework for the systematic analysis of embodied and interactive engagement that can be integrated in practice-based projects.

Another two articles examine how methods of music practice may be expanded through a focus on embodied cognition processes. As a result of a narrative review on music perception, Reybrouck and Schiavio suggest that dynamic integration of the musician's internal, sensorimotor experience and the external auditory output they perceive can support their music performance abilities and unlock potential. In a related gesture, Michałko et al. draw on sensorimotor action-perception and entrainment theory to consider how haptic information during music playing can be exchanged between humans via wearable technologies.

If Michałko et al.'s ideas for how this can be applied to practice and validated experimentally are realized, then their system might advance the dynamic integration Reybrouck and Schiavio call for to interpersonal processes across abilities and space. Here, Hansen's encouragement to build relational ethics and practice-based methods into the project design in support of diversity becomes relevant, and Shaughnessy et al.'s scoring tool for participatory engagement could be key to doing so while producing results that remain generalizable for computational use and scientific validation.

Three articles in this Research Topic aim to address concerns in the sports domain. They propose and test methods applied in novel ways to help improve the health outcomes of athletes, increase the fairness of performance assessment and support coaching strategies.

More specifically, Leguizamo et al. used qualitative interviewing and thematic coding methods to identify the subjective injury attitudes and coping mechanisms of athletes, finding both beneficial and potentially harmful approaches that highlight the need for more qualitative research into sports communication patterns. Cai and Xiang designed and tested an application of the statistical Kendall correlation coefficient method to identify inconsistencies in judges' scoring of diving competitions that may contribute to fair norms. Aiming to support basketball coaching strategy, Wang et al. developed a Bayesian Logistic Modeling approach to estimate the winning probability of a team in the fourth quarter, based on the last quarter and shooting percentage.

Thinking across these three contributions, we are invited to consider how multi-methodological consideration of health, assessment, and strategy may enable sustainable development goals for competitive sports practices and practitioners.

In combination, the seven contributions of this Research Topic indicate how Performance Science can draw on its multidisciplinary and practice-centric characteristics to develop theoretical, empirical, and applied methods empowered to solve problems and realize potentials.
